# Wine grape pomace flour improves blood pressure, fasting glucose and protein damage in humans: a randomized controlled trial

**DOI:** 10.1186/s40659-015-0040-9

**Published:** 2015-09-04

**Authors:** Inés Urquiaga, Sonia D’Acuña, Druso Pérez, Sara Dicenta, Guadalupe Echeverría, Attilio Rigotti, Federico Leighton

**Affiliations:** Centro de Nutrición Molecular y Enfermedades Crónicas, Facultad de Medicina, Pontificia Universidad Católica de Chile, Avenida del Libertador Bernardo O’Higgins 340, Santiago, Chile; Departamento de Nutrición, Diabetes y Metabolismo, Escuela de Medicina, Pontificia Universidad Católica de Chile, Santiago, Chile

**Keywords:** Antioxidant, Dietary intervention, Fiber, Metabolic syndrome, Oxidative stress, Wine grape pomace

## Abstract

**Background:**

The Mediterranean diet is a healthy diet with positive scientific evidence of preventing chronic diseases. Bioactive components support the healthy properties of the Mediterranean diet. Antioxidants and fiber, two components of the Mediterranean diet, are key functional nutrients for healthy eating and nutrition. Wine grape pomace is a rich source of these dietary constituents and may be beneficial for human health. Our hypothesis was that the intake of red wine grape pomace flour (WGPF) prepared from red wine grapes (Cabernet Sauvignon variety) reduced the metabolic syndrome in humans. To evaluate the effect of WGPF on components of metabolic syndrome we design a 16-week longitudinal intervention study. Thirty-eight males, 30–65 years of age, with at least one component of metabolic syndrome, were randomly assigned to either the intervention group (n = 25) or the control group (n = 13). At lunch, the intervention group was given 20 g of WGPF per day, which contained 10 g of dietary fiber, 822 mg of polyphenols and an antioxidant capacity of 7258 ORAC units. Both groups were asked to maintain their regular eating habits and lifestyles. Clinical evaluation, anthropometric measurements and biochemical blood analyses were done at the beginning and the end of the study.

**Results:**

WGPF intake significantly decreased systolic and diastolic blood pressure as well as fasting glucose levels. Plasma γ-tocopherol and δ-tocopherol increased and carbonyl group in plasma protein decreased in WGPT group, significantly. No significant effect was observed for waist circumference, HDL cholesterol, triglycerides, total antioxidant capacity and vitamin C in and between groups. The group-dependent magnitude of the differences between the baseline and final postprandial insulin values and γ-tocopherol concentrations was statistically significant.

**Conclusions:**

The consumption of WGPF-rich in fiber and polyphenol antioxidants, as a food supplement in a regular diet improves blood pressure, glycaemia and postprandial insulin. In addition, increased antioxidant defenses and decreased oxidative protein damage indicating attenuation of oxidative stress. WGPF might be a useful food ingredient for health promotion and chronic disease prevention.

## Background

Lifestyle changes, including healthy eating, exercise, and giving up smoking, constitute the most powerful tools to fight chronic diseases, which are the main causes of disability and death in the developed and developing world [1]. Among various conditions, patients with metabolic syndrome are at very high chronic disease risk. The increased risk associated with metabolic syndrome appears to be linked to higher levels of oxidative stress [2]. Recently, much attention has been paid to evidence that postprandial oxidative stress contributes to the development of cardiovascular disease and diabetes [3–7]. Thus, managing oxidative stress can be an important factor in managing the risk of chronic disease.

Mediterranean diets (MD) are considered as valuable preventive measures since several studies have shown that their intake is associated with lower incidence and prevalence of chronic diseases, mainly cardiovascular diseases, and longer life expectancy in the countries where a MD is consumed or in subgroups exhibiting a higher MD score [8, 9]. In 2013, the PREDIMED study, a primary prevention trial, reported the benefits of a MD in reducing the incidence of major cardiovascular events among a high-risk population [10]. A relatively large consumption of olive oil, poultry, fish, fruits, vegetables and cereals, a moderate intake of red wine, and a low consumption of red meats characterize MD.

The health effects of different diets can be explained by the positive functional properties of key nutrients. The bioactive components that apparently support the healthy properties of MD are antioxidants, fiber, mono and polyunsaturated fats, and phytochemicals [11]. Vegetables, fruits and legumes, which are rich in antioxidants and fiber, are associated with a lower incidence of metabolic syndrome and chronic diseases [12, 13]. Wine is also a source of polyphenol antioxidants and, when consumed in moderation, is associated with reduced risk of cardiovascular diseases [14].

Traditionally, dietary fiber and antioxidants are addressed separately as unrelated dietary compounds. Saura-Calixto has introduced the concept of antioxidant dietary fiber because ≈50 % of total dietary antioxidants, mainly polyphenolics, are transported along the small intestine linked to dietary fiber [15, 16]. These antioxidants are released from the fiber matrix within the colon by the action of bacterial microbiota, generating bioactive metabolites and an antioxidant environment [17]. Thus, it is proposed that the transport of dietary antioxidants through the gastrointestinal tract may be an essential function of dietary fiber [16].

Antioxidant dietary fiber is present in fruits and vegetables and derived products, such as grapes and apple pomace and mango peel powder [18]. Thus, fruit byproducts represent rich sources of dietary fiber and antioxidants with beneficial bioactive properties. In this regard, wine grape pomace flour (WGPF), made from seed and skin residues obtained from grapes during winemaking, contains high levels of phenolic compounds and dietary fiber.

In this study, we prepared flour from WGPF as a functional food ingredient to increase the daily intake of dietary fiber and bioactive antioxidant compounds. Based on the current evidence, we hypothesized that the daily intake of WGPF reduced the metabolic syndrome in adult subjects. On this basis, we conducted a longitudinal intervention study including male volunteers with at least one component of metabolic syndrome, who were randomly assigned to either the intervention group or the control group, to test the effects of dietary supplementation with this WGPF on components of metabolic syndrome, plasma antioxidants and protein damage.

## Results

### Subjects that withdrew from the study and side effect of WGPF

Thirty-eight participants completed the protocol: 13 controls and 25 subjects in the intervention group. After the initial randomization, three participants in the control group quit the study: one underwent a programmed cholecystectomy; another needed medical treatment that involved anti-inflammatory drugs to treat pain, and a third did not want to repeat blood tests. In the intervention group, six participants dropped out of the study: one had to undergo kidney surgery, two disliked blood sampling, and three declined to consume WGPF. Although the withdrawal, no statistical differences in baseline measurements were found between groups (Table 1). Also, some participants reported side effects during the period when they consumed WGPF: 7, exhibited increased intestinal gas; 2, heartburn; 2, slight episodes of constipation, 7, regularization of intestinal transit; 6, softer stools; 3, increased appetite; 2, dyspepsia; 2, gastroesophageal reflux.

**Table 1:** Baseline clinical, anthropometric, and biochemical characteristics of experimental and control groups

	Experimental	Control
Number of volunteers	25	13
Age (years)	44.5 ± 9.3	43.1 ± 8.4
Body mass index (kg/m^2^)	29.1 ± 3.9	27.9 ± 3.5
Body fat (%)	29.1 ± 5.3	26.8 ± 4.0
Waist circumference (cm)	102.7 ± 9.9	98.1 ± 7.0
Total cholesterol (mg/dL)	185.7 ± 33.9	197.8 ± 42.3
LDL cholesterol (mg/dL)	109.8 ± 28.5	120.6 ± 32.5
HDL cholesterol (mg/dL)	46.4 ± 10.7	41.2 ± 8.6
Triglycerides (mg/dL)	147.5 ± 88.2	179.8 ± 96.5
Systolic blood pressure (mmHg)	127.1 ± 11.5	122.4 ± 12.4
Diastolic blood pressure (mmHg)	79.7 ± 8.3	80.2 ± 6.6
Hemoglobin (g/dL)	15.4 ± 0.7	15.5 ± 1.0
Glucose (mg/dL)	92.7 ± 5.8	88.9 ± 5.8
Insulin (mg/dL)	12.5 ± 8.3	10.3 ± 4,6
GOT (U/L)	22.6 ± 5.8	26.2 ± 9.9
GPT (U/L)	27.5 ± 14.2	31.5 ± 12.6
ALP (U/L)	81.6 ± 16.1	84.4 ± 24.2
Bilirubin (mg/dL)	0.64 ± 0.30	0.65 ± 0.24

Values are mean ± SD. Data are tested by Students t-test. No statistical differences in baseline measurements were found between groups

*LDL* low-density lipoprotein, *HDL* high-density lipoprotein, *GOT* glutamic oxalacetic transaminase, *GPT* glutamic pyruvic transaminase, *ALP* alkaline phosphatase

### Dietary evaluation and anthropometric measurements

The Mediterranean diet score did not change significantly in any group by the end of the study. Table 2 shows the anthropometric parameters of the WGPF and control groups at baseline and after the intervention period. During the study, a statistically significant increase in body mass index (BMI) was observed in the control group (P < 0.05). No other statistically significant changes were found in this group. There were no variations in anthropometric variables in the WGPF supplemented group, including no change in BMI during the intervention period.

**Table 2:** Anthropometric characteristics in groups at baseline and 16 weeks

	Experimental		Control	
	Basal (0 weeks)	Final (16 weeks)	Basal (0 weeks)	Final (16 weeks)
Weight (kg)	86.7 ± 13.6	87.3 ± 13.4	81.1 ± 11.7	82.3 ± 11.5
Body mass index (kg/m^2^)	29.1 ± 3.9	29.3 ± 3.8	27.9 ± 3.5^a^	28.3 ± 3.6^b^
Body fat (%)	29.1 ± 5.3	28.7 ± 5.3	26.8 ± 4.0	26.3 ± 4.6
Basal metabolism (kcal)	1735 ± 399	1824 ± 179	1751 ± 171	1768 ± 163
Skeletal muscle (%)	33.0 ± 2.9	33.2 ± 2.9	34.5 ± 2.2	34.8 ± 2.5

Values are mean ± SD. Data are tested by Students t-test. Means within a group with different superscript letters are significantly different; P < 0.05 paired t-test

### Effects of WGPF on metabolic syndrome components

Table 3 shows data on components of metabolic syndrome in the WGPF and control groups at baseline and after the intervention period. After 16 weeks of supplementation with 20 g/day WGPF, systolic and diastolic blood pressure and fasting glucose levels decreased significantly (P < 0.05). Waist circumference, HDL cholesterol and triglycerides were not affected. No changes were observed in these components in the control group.

**Table 3:** Metabolic syndrome components in groups at baseline and 16 weeks

	Experimental		Control	
	Basal (0 weeks)	Final (16 weeks)	Basal (0 weeks)	Final (16 weeks)
Waist circumference (cm)	102.7 ± 9.9	102.5 ± 10.5	98.1 ± 7.0	98.8 ± 7.4
Systolic blood pressure (mmHg)	127.1 ± 11.5^a^	122.8 ± 8.5^b^	122.4 ± 12.4	120.0 ± 13.3
Diastolic blood pressure (mmHg)	79.7 ± 8.3^a^	74.4 ± 5.6^b^	80.2 ± 6.6	76.4 ± 9.3
Fasting glucose (mg/dL)	92.7 ± 5.8^a^	89.4 ± 7.9^b^	88.9 ± 5.8	86.9 ± 5.4
HDL cholesterol (mg/dL)	46.4 ± 10.7	46.5 ± 10.8	41.2 ± 8.6	42.7 ± 14.4
Triglycerides (mg/dL)	147.5 ± 88.2	153.0 ± 98.6	179.8 ± 96.5	157.4 ± 81.6
Number of positive criteria for metabolic syndrome	1.84 ± 1.62^a^	1.48 ± 1.29^b^	1.54 ± 1.13	1.54 ± 1.13
Metabolic syndrome prevalence (%)	32	24	23	23

Values are mean ± SD. Student *t*-test for paired samples was used to analyze differences of means within groups. Student *t*-test for independent samples was used to analyze differences of means between groups. Wilcoxon signed rank-sum test was used to compare the number of positive criteria for metabolic syndrome. Means within a group with different superscript letters are significantly different; P < 0.05 paired t test

In the control group, initially 23 % (3/13) had abdominal obesity, 54 % (7/13) high triglycerides, 31 % (4/13) low HDL cholesterol, 38 % (5/13) high blood pressure, 8 % (1/13) high glucose and 23 % (3/13) were diagnosed with metabolic syndrome at baseline. After the 16-week intervention, 31 % (4/13) of control subjects showed abdominal obesity, 38 % (5/13) high triglycerides, 54 % (7/13) low HDL cholesterol, 31 % (4/13) high blood pressure, and 0 % high glucose. During the study, no significant changes were observed in percentages of subjects presenting metabolic syndrome criteria in the control group, 23 % (3/13).

In the WGPF supplemented group, 52 % (13/25) presented abdominal obesity, 36 % (9/25) high triglycerides, 24 % (6/25) low HDL cholesterol, 52 % (13/25) high blood pressure, 20 % (5/25) high glucose, and 32 % (8/25) were diagnosed with metabolic syndrome at baseline. After the 16-week intervention, 52 % (13/25) exhibited abdominal obesity, 40 % (10/25) high triglycerides, 28 % (7/25) low HDL cholesterol, 20 % (5/25) high blood pressure, 8 % (2/25) high glucose and 24 % (6/25) had metabolic syndrome. After intake of WGPF, the percentage of subjects with high blood pressure according to the metabolic syndrome definition had decreased significantly (P = 0.035).

Overall, WGPF significantly reduced the average number of metabolic syndrome components present per subject from 1.84 ± 1.62/participant to 1.48 ± 1.29/participant (P < 0.05). No change was observed in the control group.

With regard to the diagnosis of metabolic syndrome in the control group, the prevalence of this risk condition remained unchanged (23 %) during this study. In contrast, a non-significant trend to a lower prevalence of metabolic syndrome was detected from baseline (32 %) to after WGPF supplementation (24 %).

### Effects of WGPF on additional metabolic factors

Total cholesterol and LDL cholesterol did not change significantly within and between groups by the end of the study. Table 4 shows findings from an oral glucose tolerance test (OGTT) with glucose, insulin measurements, insulin resistance (evaluated by HOMA) and glycosylated hemoglobin in the WGPF and control groups at baseline and after the intervention. As mentioned above, a statistically significant decrease was observed in fasting glucose in the WGPF supplemented group, but not in the control group (Table 3). After the 16-week intervention, there were no statistically significant changes in fasting insulin, HOMA, postprandial glucose, insulin and glycosylated hemoglobin between the two groups. While postprandial insulin levels increased from the baseline to the end of the study among control subjects, levels decreased among the members of the WGPF group over the same period. The group-dependent magnitude of the differences between the initial and final postprandial insulin values was statistically significant (P < 0.05).

**Table 4:** Oral glucose tolerance test, HOMA and glycosylated hemoglobin in groups at baseline and 16 weeks

	Experimental		Control	
	Basal (0 weeks)	Final (16 weeks)	Basal (0 weeks)	Final (16 weeks)
Postprandial glycemia 120 min (mg/dL)	85.0 ± 28.9	89.1 ± 34.2	82.7 ± 19.1	83.7 ± 34.8
Fasting insulin (mg/dL)	12.5 ± 8.3	11.8 ± 8.9	10.3 ± 4.6	8.9 ± 4.4
Postprandial insulin 120 min (mg/dL)	67.1 ± 68.3	60.3 ± 59.6*	57.8 ± 57.0	77.0 ± 97.2*
HOMA	2.90 ± 2.05	2.65 ± 2.17	2.30 ± 1.10	1.93 ± 1.02
Glycosylated hemoglobin (%)	5.2 ± 0.3^a^	5.5 ± 0.3^b^	5.1 ± 0.3^a^	5.3 ± 0.3^b^

Values are mean ± SD. Student t-test for paired samples was used to analyze differences of means within groups. Student t-test for independent samples was used to analyze differences of means between groups. Means within a group with different superscript letters are significantly different; P < 0.05 paired t test

* Indicate statistically significant differences in the changes produced by the intervention between the groups (P < 0.05, paired t test)

### Effects of WGPF on antioxidant vitamins, antioxidant capacity and oxidative damage in proteins

Table 5 shows data on plasma tocopherols, vitamin C, antioxidant capacity and carbonyl groups in proteins, in the WGPF and control groups at baseline and after the intervention period. Alpha-tocopherol and antioxidant capacity measure as TRAP did not change significantly within and between groups by the end of the study. Vitamin C increased and antioxidant capacity, measure as DPPH, decreased from the baseline to the end of the study within control and WGPF groups significantly. After the 16-week intervention γ-tocopherol and α-tocopherol increased significantly in WGPF group. Additionally, while δ-tocopherol concentrations increased from the baseline to the end of the study among the members of the WGPF group, concentrations decreased among control subjects over the same period. The group-dependent magnitude of the differences between the initial and final γ-tocopherol values was statistically significant (P < 0.05). Protein damage measured as carbonyl groups in plasma proteins decreased significantly in WGPF group by the end of the study.

**Table 5:** Antioxidant vitamins, antioxidant capacity and protein carbonylation in groups at baseline and 16 weeks

	Experimental		Control	
	Basal (0 weeks)	Final (16 weeks)	Basal (0 weeks)	Final (16 weeks)
α-Tocopherol (µM)	31.67 ± 8.58	32.48 ± 8.73	33.00 ± 10.12	31.75 ± 9.85
γ-Tocopherol (µM)	1.80 ± 0.74^a^	2.40 ± 1.36^b^*	1.97 ± 1.07	1.87 ± 0.72*
δ-Tocopherol (µM)	0.70 ± 0.13^a^	0.79 ± 0.23^b^	0.74 ± 0.26	0.76 ± 0.24
Vitamin C (µM)	28.66 ± 8.41^a^	34.58 ± 11.76^b^	23.16 ± 8.47^a^	30.21 ± 10.12^b^
TRAP (µM Trolox Eq.)	491.99 ± 114.69	469.82 ± 105.74	465.92 ± 82.97	424.96 ± 71.65
DPPH (µM Trolox Eq.)	93.47 ± 28.11^a^	72.05 ± 26.97^b^	91.04 ± 24.82^a^	67.16 ± 22.49^b^
Carbonyl groups (nmol/mg protein)	0.56 ± 0.18^a^	0.44 ± 0.19^b^	0.55 ± 0.14	0.54 ± 0.29

Values are mean ± SD. Student t-test for paired samples was used to analyze differences of means within groups. Student t-test for independent samples was used to analyze differences of means between groups. Means within a group with different superscript letters are significantly different; P < 0.05 paired t test

* Indicate statistically significant differences in the changes produced by the intervention between the groups (P < 0.05, paired t test)

## Discussion

Wine grape pomace flour prepared from red wine grape, Cabernet Sauvignon variety, is especially rich in antioxidants and fiber, two bioactive components of human diet with healthy properties. Our hypothesis was that WGPF consumption decreases the risk of developing metabolic syndrome, supporting its potential benefit for preventing cardiovascular disease and diabetes. We chose a group of men with at least one diagnostic criterion of metabolic syndrome in order to enhance the chance of detecting significant effects.

WGPF contains an antioxidant/fiber complex, in which polyphenols are transported along the gastrointestinal tract linked to fiber components. WGPF includes soluble or extractable polyphenols with low or intermediate molecular mass, which appear to be absorbed from the digestive tract with systemic effects [15]. Nevertheless, many WGPF polyphenols are non-absorbable/non-extractable high molecular mass proanthocyanidins [16]. After reaching the large intestine, these antioxidants are processed by colonic microbiota producing metabolites with physiological properties [19].

Antioxidant dietary fiber and extractable and non-extractable polyphenol content in pomace obtained during winemaking usually ranges from 50 to 75 %, 1 to 9 % and 15 to 30 % by dry matter, respectively [15]. Our WGPF contains 52 % dietary fiber (7 % soluble and 93 % insoluble), and 4.4 % of extractable polyphenols with an antioxidant capacity of 362.9 ORAC (µmol TE/g dry matter). A similar product obtained from Cencibel wine grape in Spain showed an antioxidant capacity of 214.2 ORAC units [20]. Thus, flour derived from Cabernet Sauvignon WGPF exhibits a composition profile that suggests potential health benefits.

Indeed, dietary fiber (or non-starch polysaccharides) is a collective term for a variety of plant substances resistant to digestion by human gastrointestinal enzymes. Structural fiber (cellulose, lignin, and hemicelluloses) is insoluble, whereas gel-forming fiber (pectins, gums, and mucilages) is water-soluble [21]. Intervention trials have shown that soluble dietary fiber lowers blood cholesterol concentrations, reduces blood pressure, promotes body weight loss, and improves insulin sensitivity, eventually leading to reduced risk of cardiovascular disease [21, 22].

Based on proximal analysis of WGPF (Table 6), supplementation with 20 g of WGPF increased the intake of dietary fiber by 9.5 g per day. Previous data from a similar group of workers disclosed a consumption of 19.5 g/day of dietary fiber [23]. Thus, we estimated that intake of fiber might have reached ≈30 g/day in the WGPF supplemented group during the study. In addition, this amount of WGPF provided 7,258 ORAC units/day of antioxidant capacity, which is equivalent to consuming two glasses of red wine per day.

**Table 6:** Composition of wine grape pomace flour

	g/100 g WGPF
Proximal analysis and fiber	
Fat	7.75
Protein	11.71
Carbohydrates^a^	16.96
Dietary fiber	47.70
Soluble	3.54
Insoluble	44.20
Ash	8.41
Moisture	7.47
Antioxidants and antioxidant capacity	
Polyphenols (mg GE/g)	41.11 ± 3.01
Anthocyanin (mg C3G/g)	1.49 ± 0.18
Vitamin C (µg/g)	n.d.
α-tocopherol (µg/g)	53.51 ± 3.69
γ-tocopherol (µg/g)	12.57 ± 0.71
δ-tocopherol (µg/g)	0.68 ± 0.07
ORAC (µmoles TE/g)	362.9 ± 24.4

Values are mean ± SD

*GE* gallic equivalent, *C3G* cyanidin 3-glucoside, *TE* Trolox equivalent, *n.d.* non-detected

^a^Nitrogen-free extract minus dietary fiber

We observed a statistically significant lowering of blood pressure after WGPF intake. Previous studies have shown that soluble dietary fiber reduces blood pressure [21, 22]. Furthermore, fiber, especially soluble types, also improves intestinal potassium and magnesium absorption, which may have an indirect favorable effect on blood pressure [22]. However, the WGPF used in this study contains only 7 % of soluble fiber. Consequently, it is difficult to attribute the hypotensive effect exclusively to this component. Nevertheless, extracts of polyphenols from grape seeds, grape pomace, and/or wine have shown positive effects on blood pressure. Polyphenols activate endothelial nitric oxide synthase (eNOS) and this effect has been suggested as the mechanism that lowers blood pressure [24]. The bioavailability of single and 2- or 3-polymer unit polyphenols also support this mechanism [25].

A relatively small fraction (1–9 %) of these polyphenols associated with dietary fiber are extractable from WGPF [15], so only a minor amount of this form of antioxidants are absorbed by the intestine. However, plasma antioxidant capacity increases postprandially, becoming significant 8 h after consuming grape pomace [26]. This delayed increase is explained by the presence of more complex polyphenols (high polymerization proantocyanidins) linked to fiber in grape pomace. These proantocyanidins reach the colon, where they are partially fermented by bacterial microflora along with indigestible carbohydrates. Indeed, colonic fermentation of grape pomace generates metabolites that are detectable in human plasma [17]. Fiber-associated antioxidant products released by fermentation may explain the reduction in blood pressure after WGPF supplementation. Additional studies are required to test this hypothesis.

Moreover, we observed a significant reduction in the plasma concentrations of fasting glucose in the WGPF supplemented group. There is evidence that dietary fiber consumption is associated with lower fasting glucose levels in diabetic individuals [27]. The mechanisms associated with this beneficial effect have not yet been elucidated [28].

We also found, a statistically significant difference, when comparing the change in postprandial insulin concentrations along the study, among control and WGPF groups. There have been few intervention studies on the effects of dietary fiber on postprandial glucose and insulin. Recently, an intervention trial reported that a whole-grain cereal-based diet significantly lowers postprandial plasma insulin concentration in individuals with metabolic syndrome [29]. It is likely that bioactive compounds in this fiber-enriched ingredient, such as trace minerals, phenolic antioxidants, vitamin E or phytoestrogens, operating by different unknown pathways may contribute to this positive postprandial metabolic effect [30].

In prospective observational studies, normal postprandial increases in blood glucose and triglyceride levels have been linked to the risk of cardiovascular disease and diabetes [3–7]. The evidence suggests that postprandial hyperglycemia and hypertriglyceridemia cause oxidative stress. Postprandial oxidative stress occurs within minutes to hours following consumption of a meal rich in fat, carbohydrates and protein [31, 32]. Over the long-term, oxidative stress leads to insulin resistance, pancreatic β-cell dysfunction, abnormal serum lipids, coagulopathy, systemic inflammation, and endothelial dysfunction [33]. Exaggerated postprandial spikes in glucose and lipids can also trigger inflammation, endothelial dysfunction, and sympathetic hyperactivity [33]. With multiple repetition on a daily basis, these postprandial changes that lead to atherosclerosis and diabetes. Dietary strategies aimed at attenuating these pathogenic alterations provide new opportunities to reduce the risk of chronic disease. In fact, consumption of red wine polyphenols and grape seed extract with a meal rich in fat, carbohydrates and protein significantly reduces postprandial oxidative stress [34–36]. We hypothesize that dietary fiber rich in antioxidant in our WGPF reduces postprandial oxidative stress and plasma oxidative damage when consumed with meals. In accord, we observed a significant decrease in protein oxidation in WGPF supplemented group. Oxidative damage reduction may partially explain its beneficial effect on blood pressure and glucose homeostasis, with important implications for the prevention and management of metabolic syndrome [24]. Further investigation is needed to validate this proposal.

In contrast to earlier studies with pomace with animal models and humans [16, 20, 37, 38], our WGPF did not influence serum cholesterol levels. In a human trial, supplementation with grape pomace flour obtained from Cencibel wine grapes significantly decreased total cholesterol in normo- and hypercholesterolemic subjects [20]. Furthermore, LDL cholesterol and triglycerides diminished significantly only in hypercholesterolemic subjects, who represented 38 % of study participants and were mostly women (65 %) [20]. Although the fiber and antioxidant composition and dose were similar to those in our WGPF, we did not observe any effect on lipid profile. However, our study design included only male subjects with 40 % hypercholesterolemic (total cholesterol >200 mg/dL) at baseline. Alternatively, the lack of effect in our study may be attributed to differential functional properties of wine grape types (e.g., Cabernet Sauvignon versus Cencibel). In hamsters, different effects were observed of a diverse variety of grape pomace in plasma total cholesterol and LDL-cholesterol concentrations [39].

In terms of antioxidant capacity, measured as DPPH, and vitamin C, we observed similar changes in both group. They consumed same food at the cafeteria of work place, so these variations could be explained by the diet.

Interestingly we found a significant increase in plasma concentrations of γ-tocopherol and δ-tocopherol in the WGPF supplemented group. Also, a statistically significant difference, when comparing the change in γ-tocopherol concentrations along the study, among control and WGPF groups was detected. The raise in plasma concentrations of γ- and δ-tocopherols in the WGPF supplemented group could be explained by the antioxidant effect of WGPF polyphenols in the gastrointestinal tract [40]. This effect would depend on the quantity of tocopherols in the entire diet. Volunteers consumed soybean oil that is a good source of γ-tocopherol (80 mg/100 g) and δ-tocopherol (27 mg/100 g) [41]. WGPF by its antioxidant capacity would protect these vitamins from its oxidation, improving its bioavailability and increasing the antioxidant defenses of the volunteers in terms of plasma concentration of vitamin E.

The significance of plasma increase of these forms of vitamin E on health is matter of discussion [41]. Most research of vitamin E has focused on α-tocopherol, because it is the predominant form of vitamin E in tissues. Despite well-documented antioxidant and other beneficial effects, supplementation with α-tocopherol has failed to offer consistent benefits to prevention of chronic diseases, as cancer and cardiovascular diseases, in clinical intervention studies in people with adequate nutrient status. Current studies show that other forms of vitamin E, such as γ-tocopherol and δ-tocopherol have unique antioxidant and anti-inflammatory properties in prevention and therapy against chronic diseases. These vitamin E forms scavenge reactive nitrogen species, inhibit cyclooxygenase- and 5-lipoxy-catalyzed eicosanoids, and suppress proinflammatory signaling. It has been suggested that γ-tocopherol and δ-tocopherol may be useful against inflammation-associated diseases [41].

Strength of the current study was that workers had a similar diet during weekdays, because they were offered same lunch menus at the cafeteria of the company. Also we supervised daily the intake of WGPF during lunch as well as similar consumption of regular bread in control subjects. However, one limitation of the study was that the number of participants who completed the protocol in each group was relatively small, because six and three participants quit the study in WGPF and control groups, respectively. Second, this study was an open-labelled trial that may have led to potential biases in the subjects as well as in the researchers. Third, we did not measured differences in caloric intake at the end of the interventions, but no changes in abdominal obesity or body weight were detected during the study in both groups suggesting that no major differences in energy balance occurred during the study. In addition, both groups were encouraged to maintain their regular eating habits and lifestyle.

## Conclusions

The consumption of WGPF as a food supplement to a regular diet, improves some components of metabolic syndrome, as blood pressure and glycaemia. Also, postprandial insulin levels, measured in an OGTT, were reduced. Moreover, fiber and polyphenols-containing WGPF increased antioxidant defenses, rising γ- and δ-tocopherols and diminishing oxidative protein damage, contributing to a better handling of oxidative stress. WGPF seems to be a promising functional ingredient that can be incorporated into different food matrices to increase fiber and antioxidant consumption and eventually contribute to the management of chronic disease burden.

## Methods

### Subjects

A group of 47 male workers 30–65 years old, who regularly consumed an omnivorous diet, participated in the study. All of them gave informed consent and underwent a comprehensive medical history, physical examination, and clinical chemistry analysis before enrollment. One of the inclusion criteria was having at least one of the five components of metabolic syndrome.

The presence of metabolic syndrome components was defined using the criteria proposed by the Adult Treatment Panel III of the US National Cholesterol Education Program: (1) abdominal obesity as waist circumference >102 cm for men; (2) low levels (<40 mg/dL for men) of serum high density lipoprotein cholesterol; (3) hypertriglyceridemia as 150 mg/dL or more; (4) elevated blood pressure as 130/85 mmHg or higher; and (5) impaired glucose homeostasis as fasting plasma glucose levels of 100 mg/dL or higher [42].

Volunteers were excluded that were under treatment for diabetes mellitus, hypertension or dyslipidemia or under pharmacological treatment with drugs that modify plasma antioxidant capacity and inflammation. The study was approved by the Ethics Committee of the School of Medicine at the Pontificia Universidad Católica de Chile and was performed in accordance with the ethical standards laid down in the 1964 Declaration of Helsinki and Michael Okpara University, Umudike, Nigeria.

### Wine grape pomace flour

Pomace, a byproduct from wine production, was obtained from red wine grapes (Cabernet Sauvignon, vintage 2011, Maipo Valley, Chile) and stored at −20 °C until used. Frozen WGPF was thawed at room temperature and dried in a forced air dryer at 60 °C until moisture reached less than 12 %. Dried pomace was powdered into flour using a hammer mill. WGPF was packaged in 20-kilo double plastic bags.

The overall composition of WGPF is shown in Table 6. It has a high dietary fiber content (52 % by dry weight) determined by the enzymatic–gravimetric method AOAC 991.43. In addition, WGPF was sequentially extracted three times with acetone/water/acetic acid at room temperature for 60 min and supernatants were combined and used to measure total polyphenol content, antioxidant capacity and tocopherols. Polyphenolic compounds (41.11 mg gallic acid equivalent/g) were determined by the Folin–Ciocalteu procedure [43]. Total anthocyanin content (1.49 ± 0.18 mg/g cyanidin 3-glucoide equivalents) was determined by AOAC Official Method 2005.02. Antioxidant capacity (362.9 µmoles/g Trolox equivalent) was measured by the oxygen radical absorbance capacity (ORAC) assay [44]. There was not detected vitamin C (l-ascorbic acid) analyzed using the HPLC method described by Kimoto et al. [45]. Tocopherol contents (α-tocopherol 53.51 ± 3.69 µg/g; γ-tocopherol 12.57 ± 0.71 µg/g and δ-tocopherol 0.68 ± 0.07 µg/g) were determined using high-performance liquid chromatography (HPLC) with electrochemical detection according to Motchnik et al. [46].

Quantification and identification of polyphenols and anthocyanins in WGPF extracts were performed by HPLC. The HPLC chromatogram of polyphenol neutral fraction with detection at 360 nm, the HPLC chromatogram of polyphenol acid fraction with detection at 280 nm and the HPLC chromatogram of polyphenol aqueous fraction (anthocyanin fraction) with detection at 518 nm. Individual compounds were identified by diode array detector (DAD) and standard compounds. Phenolic acids (gallic acid; caffeic acid; vanillic acid; protocatechuic acid; coumaric acid and ferulic acid) concentration was 0,065 mg/g WGPF, in which gallic acid represented 83 %. Flavonoids (flavanols; flavonols and flavanones) concentration was 0.160 mg/g WGPF, in which catechin and catechin derivatives represented 57.8 % and flavonols 22.9 %. Anthocyanin concentration was 0.366 mg/g WGPF (mono-glucosides were expressed as cyanidin-3-glucoside and di-glucosides as cyanidin-3,5-diglucoside), in which malvidin and malvidin derivatives represented 65 %.

### Study design

The study was a prospective, randomized, controlled parallel-group trial. The intervention was carried out at a heavy mining machinery company in Santiago, Chile. All employees were informed about the study and invited to participate. Initially, 47 male workers free of exclusion criteria agreed to participate. The number of participants in each group was chosen based on the variation coefficients in the evolution of total cholesterol in the intervention and in the control group, observed in a previous work [47].

For allocation of the participants, a computer-generated random list was used, prepared by an investigator with no clinical involvement in the trial. Participants were randomly assigned to either the control (n = 16) or intervention group (n = 31). Both groups were asked to maintain their regular eating habits and lifestyles for 16 weeks, except for the daily intake of 20 g of WGPF by the intervention group. WGPF was consumed in bread, biscuits or as flour mixed with water during lunch. Bread and biscuits with 20 % WGPF were prepared especially in a bakery. WGPF intake was supervised every day at lunch. Participants were asked to consume the flour supplement with their regular meals on weekends.

Complete follow-up was obtained with a group of 38 workers, 13 controls and 25 subjects that consumed WGPF. Compliance with WGPF consumption was carefully monitored. Participants had clinical, nutritional, anthropometric and laboratory evaluations at the beginning and end of the study. There were no significant basal differences between the two groups in anthropometric, blood pressure and biochemical parameters (Table 1).

### Mediterranean diet score

Overall food intake was evaluated using a self-reported questionnaire with fourteen items that measure adherence to the Mediterranean diet in Chile [23]. This scale was designed on the basis of traditional food consumption habits in the European Mediterranean region, with selective modifications to incorporate Chilean dietary habits. This Mediterranean diet score ranges from 0 (minimal adherence) to 14 (maximal adherence). There were no significant basal differences between the two groups in the Mediterranean diet score, with a score of 5.0 ± 1.32 in the control group and 5.4 ± 1.56 in the supplemented group.

### Anthropometric and blood pressure measurements

Height, weight, and percentage of body fat (determined by bioimpedance, Omron device model HBF-500INT) were recorded at baseline and at the end of the intervention period.

Blood systolic and diastolic pressures were measured on the left arm at the heart level after at least 5 min of resting in a sitting position and using a mercury sphygmomanometer (RIESTER Diplomat^®^-Presameter). Two readings, separated by at least 1 min, were taken and the mean value was calculated and recorded. If there was more than a 5 mmHg difference between the first and second readings, additional readings were obtained and then the mean value of the multiple readings was used [48].

### Biochemical procedures

Venous blood samples were taken after a 12-h fasting period and collected in heparin, citrate and anticoagulant-free BD Vacutainer^®^ tubes. Glucose, total cholesterol, HDL cholesterol, LDL cholesterol and triglycerides were measured in serum using a spectrophotometer autoanalyzer (Hitachi 917; Roche Diagnostics^®^) with reagent kits purchased from the manufacturer.

An OGTT was performed after overnight fasting. Subjects ingested a solution containing 75 g of dextrose, and venous blood samples were obtained at 0 and 120 min to determine plasma glucose and insulin levels. Insulin was measured by electrochemiluminescence immunoassay (ECLIA; Roche Diagnostics^®^).

For determination of l-ascorbic acid, blood samples kept in ice were analyzed the same day that blood was drawn. l-Ascorbic acid was determined by spectrophotometry [46].

Tocopherols (α-tocopherol, γ-tocopherol and δ-tocopherol) were determined in plasma using HPLC with electrochemical detection according to Motchnik et al. [46].

The total plasma antioxidant capacity was evaluated by total radical trapping potential (TRAP) and 2,2-diphenyl-1-picrylhydrazyl (DPPH) assays. Trolox^®^ (6-hydroxy-2,5,7,8-tetramethylchroman-2-carboxylic acid) (Trolox),was chosen as a standard antioxidant. The procedure for TRAP employs luminol-enhanced chemiluminescence measurements, method developed by Wayner et al. [49]. DPPH assay was assessed by the spectrophotometric method adapted from Pisoschi et al. [50].

Determination of carbonyl groups in oxidized plasma proteins was assessed by derivatization with dinitrophenylhydrazine (DNPH). Spectrophotometric measurement of plasma reactive carbonyl derivatives was performed and calculated using the extinction coefficient of DNPH-reactive carbonyl derivatives at 370 nm = 22 × 103 L mol^−1^ cm^−1^ and expresses as μmol/g proteins Levine et al. [51].

### Statistical analysis

Continuous variables are shown as mean and standard deviation, while categorical variables as the number of cases and percentage. The student t-test for independent samples and the Chi square test were used to analyze differences of means and proportions between the two groups, respectively. The student t-test for paired samples and McNemar’s test were used to analyze differences of means and proportions within each group. When appropriate, the Wilcoxon signed rank-sum test was used to compare paired medians. All P values were two-tailed and a value <0.05 was considered to be statistically significant. Data processing and statistical analyses were done with the SAS statistical software package version 9.1 for Windows.

## Abbreviations

ALP: alkaline phosphatase
AOAC: association of Official Agricultural Chemists
C3G: cyanidin 3-glucoside
DAD: diode array detector
BMI: body mass index
eNOS: endothelial nitric oxide synthase
GE: gallic equivalent
GOT: glutamic oxalacetic transaminase
GPT: glutamic pyruvic transaminase
HDL: high-density lipoprotein
HOMA: homeostasis model assessment
HPLC: high pressure liquid chromatography
LDL: low-density lipoprotein
MD: Mediterranean diet
WGPF: wine grape pomace flour
OGTT: oral glucose tolerance test
ORAC: oxygen radical absorbance capacity
SD: standard deviation
TE: Trolox equivalent
